# Surveillance of Transmitted Antiretroviral Drug Resistance among HIV-1 Infected Women Attending Antenatal Clinics in Chitungwiza, Zimbabwe

**DOI:** 10.1371/journal.pone.0021241

**Published:** 2011-06-16

**Authors:** Mqondisi Tshabalala, Justen Manasa, Lynn S. Zijenah, Simbarashe Rusakaniko, Gerard Kadzirange, Mary Mucheche, Seble Kassaye, Elizabeth Johnston, David Katzenstein

**Affiliations:** 1 Department of Immunology, University of Zimbabwe College of Health Sciences, Harare, Zimbabwe; 2 Department of Community Medicine, University of Zimbabwe College of Health Sciences, Harare, Zimbabwe; 3 Department of Medicine, University of Zimbabwe College of Health Sciences, Harare, Zimbabwe; 4 Department of Medicine, Stanford University School of Medicine, Stanford, California, United States of America; University of California, San Francisco, United States of America

## Abstract

The rapid scale-up of highly active antiretroviral therapy (HAART) and use of single dose Nevirapine (SD NVP) for prevention of mother-to-child transmission (pMTCT) have raised fears about the emergence of resistance to the first line antiretroviral drug regimens. A cross-sectional study was conducted to determine the prevalence of primary drug resistance (PDR) in a cohort of young (<25 yrs) HAART-naïve HIV pregnant women attending antenatal clinics in Chitungwiza, Zimbabwe. Whole blood was collected in EDTA for CD4 counts, viral load, serological estimation of duration of infection using the BED Calypte assay and genotyping for drug resistance. Four hundred and seventy-one women, mean age 21 years; SD: 2.1 were enrolled into the study between 2006 and 2007. Their median CD4 count was 371cells/µL; IQR: 255–511 cells/µL. Two hundred and thirty-six samples were genotyped for drug resistance. Based on the BED assay, 27% were recently infected (RI) whilst 73% had long-term infection (LTI). Median CD4 count was higher (p<0.05) in RI than in women with LTI. Only 2 women had drug resistance mutations; protease I85V and reverse transcriptase Y181C. Prevalence of PDR in Chitungwiza, 4 years after commencement of the national ART program remained below WHO threshold limit (5%). Frequency of recent infection BED testing is consistent with high HIV acquisition during pregnancy. With the scale-up of long-term ART programs, maintenance of proper prescribing practices, continuous monitoring of patients and reinforcement of adherence may prevent the acquisition and transmission of PDR.

## Introduction

International guidelines recommend genotypic drug resistance testing among patients initiating antiretroviral therapy (ART) [1], [2], [3], [4]. However, this is neither affordable nor practical in many resource limited settings implementing a public health approach to ART, with standardized low cost first line ARV regimens and minimal laboratory monitoring. The efficacy of first line regimens and long term sustainability of ART may be limited by the emergence of drug resistance [5]. Recent models of infection in Africa suggest that a decrease in population virus burden due to increased access to ART coupled with a diminished transmissibility of drug resistant viruses could decrease HIV incidence [6]. However, public health and patient benefit may be limited by an increase in risky behaviors and transmission of drug resistant viruses [7].

PDR has been documented in European and US studies of recent and acute infection. In these studies, prevalence of PDR ranges from 6% to 23%. [8], [9], [10], [11], [12], [13], [14]. A large study of ART naïve HIV-infected individuals from 40 United States cities reported a prevalence of drug resistance associated mutations of 12% among chronically infected, newly diagnosed individuals [15]. In Africa, potential contributors to the emergence and transmission of drug resistant HIV are intermittent drug supplies, inadequate patient monitoring, incorrect prescribing practices, variable adherence and the use of SD NVP in pMTCT programs [16], [17]. Several surveillance studies in Africa estimate the prevalence of transmitted drug resistance to be less than 5% [18], [19], [20], [21], [22], [23]. However, a few studies in East and Southern Africa have recently reported evidence of increasing levels of transmitted drug resistance [24], [25].

Systematic drug resistance surveillance to monitor PDR is recommended in countries scaling-up ART [26]. As part of monitoring the HIV epidemics, it is also critical to assess PDR in the newly HIV-infected individuals. Longitudinal cohort studies for estimation of new infections are prohibitively expensive. A number of alternative methods have been developed. The Calypte HIV-1 BED Incidence Enzyme Immunosorbent Assay (cBED) assay [27] is the most widely used. The assay identifies infection within the last 6 months based on the increasing proportion of anti-HIV IgG in total IgG following sero-conversion [28] and is recommended by CDC for population-based incidence studies [27]. For more accurate estimates of incidence, cBED assay in different subtypes and contexts, results may be adjusted using population specific false positive rates (FPR) to provide estimates consistent with longitudinal studies [29], [30], [31]. There were concerns that the cBED assay in pregnant women may overestimate recent infection because of haemodilution and immune suppression of pregnancy, which could delay the maturation of antibodies. However recent evidence suggest that pregnancy does not affect the cBED results [32].

We conducted systematic drug resistance surveillance among young HIV-1 sero-positive pregnant women over a period of two years (2006–2007) and performed cBED assays to estimate the frequency of recent infections. The aim of the study was to characterize contemporary, recently acquired HIV-1 Subtype C drug resistance mutations among young pregnant women in Zimbabwe.

## Materials and Methods

Four hundred and seventy-one HIV-1 antibody positive young pregnant women attending antenatal clinics in Chitungwiza were enrolled over a period of two years (2006–2007). Chitungwiza, the third largest urban center in the country, is located about 25 kilometers south of Harare, the Capital of Zimbabwe. A comprehensive demographic questionnaire was administered to determine if the participants or their sexual partner(s) had exposure to ART. This was done in order to distinguish acquired/transmitted drug resistance from drug resistance attributable to ARV use. The study protocol and the questionnaire used were reviewed and approved by the Medical Research Council of Zimbabwe, the Research Council of Zimbabwe and the Stanford University Institutional Review Board. Signed informed consent was obtained from all participants.

Whole blood was collected in EDTA for enumeration of CD4^+^ T lymphocytes, virus load, detection of drug resistance mutations and estimation of the duration of infection. CD4^+^ T lymphocytes were enumerated using a Partec Cyflow counter (Cyflow, Partec, Munster, Germany) within 6 hours of blood collection as previously described [33], [34]. The remaining whole blood was centrifuged and plasma was aliquoted and stored at −80°C.

HIV-1 RNA levels were quantified using the standard Roche Amplicor Version 1.5 assay (Roche Molecular Sciences, Pleasanton CA, USA) in Zimbabwe. Aliquots of plasma were shipped to Stanford University using dry nitrogen shippers. Upon receipt they were stored at −80°C until sequenced. For sequencing, the samples were thawed and ultra centrifuged at 23000 g for 45 minutes at −4°C to pellet virus particles. RNA was extracted from the viral pellets using a commercial RNA extraction kit (Roche Molecular Diagnostics, NJ), reverse transcribed to cDNA using random hexamers and the reverse transcriptase enzyme Superscript III (Invitrogen Corporation, Carlsbad CA). A 1315 bp fragment of the pol gene covering 99 protease codons and the first 240 codons of the reverse transcriptase (RT) was amplified using Platinum Taq (Invitrogen Corporation, Carlsbad CA) polymerase by nested PCR and sequenced as previously described [35].

Sequences were assembled using Sequencher Version 4.8 (Gene Codes Corporation, Ann Arbor, MI) and analyzed using the Stanford HIVDB's QA/QC program which compares new sequences to other sequences produced from the same laboratory to identify possible contamination. To aid in quality assurance, neighbor joining phylogenetic trees were also created using Bioedit version 7.0.0 (Ibis Biosciences, Carlsbad CA) for sequence alignments and MEGA version 4.0.2 (The Biodesign Institute, Tempe AZ) for neighbor-joining tree generation. Maximum Likelyhood (ML) trees were generated in PhyML v 2.4.4 [36], and 1000 replicates were bootstrapped. The sequences were then analyzed by the REGA HIV-1 Subtyping tool [37] on the Stanford HIV Data Base (HIVDB) for subtype identity and to evaluate for inter-subtype recombination. The Stanford HIVDB HIVSEQ algorithm was used to check for known drug resistance mutations. HIVDB's Calibrated Sequences Program was used to evaluate the sequences for transmitted drug resistance. The aligned sequences in Bioedit software were used to generate the Zimbabwean HIV-1 Subtype C pol consensus sequence.

The cBED assay was conducted according to the manufacture's instructions (Calypte Biomedical Corporation, OR). In brief, HIV-specific IgG were detected in 1:101 diluted plasma samples by the BED-biotin peptide, followed by a colour reaction with streptavidin-peroxidase. The optical density values were normalized in every run using a calibrator (normalized OD (ODn)  =  mean specimen OD/mean calibrator OD). Specimens with ODn less than or equal to 1.2 during an initial cBED screening test were confirmed by further cBED testing of the sample in triplicate. The median value of the three confirmatory test results was used as the final ODn value. HIV-1-positive specimens for which the cBED assay gave a final ODn of less than or equal to 0.8 were classified as recent HIV-1 infection.

The statistical program STATA version 10 (StataCorp LP, Texas, USA) was used to perform Chi squared tests for categorical data and the Student T test for continuous data.

## Results

Four hundred and seventy-one pregnant women with a mean age of 21.5 years (SD: 2.1) participated in this study. Of these, 53.8% (95% CI: 49.3; 58.5) were primigravida. The proportion of women who reported more than one sexual partner in the past 12 months was 17. 4% (95% CI: 13.9–20.9) and 22.9% (19.0–26.8) had been previously treated for a sexually transmitted infection (STI). The mean age of their partners was 28.7 years (SD: 5.8). The median CD4 count of the 471 women was 371 cells/µL (IQR: 255–511).

Based on the results of the cBED assay from the 470 women, 27% (125 of 470) were classified as likely to have been infected within 155 days of sampling or recently infected (RI) and 330 (73%) as long-term infection (LTI). Table 1 summarizes the comparison of clinical, demographic and laboratory characteristics of the RI and LTI.

**Table 1:** Patient demographic data grouped according to their infection duration based on the BED results.

Demographic information	All women	Long Term Infection	Recently Infected	p-value
Age, women, mean (SD), years	21.5 (2.1)	21.6 (2.0)	21.2 (2.2)	0.059
Age of husband, mean (SD), years	28.7 (5.8)	28.5 (4.7)	29.5 (5.0)	0.048
Age at 1st menses, mean (SD), years	14.4 (1.6)	14.5 (1.6)	14.2 (1.4)	0.035
Proportion ever been forced to have sex (95% CI)	5.9% (3.8;8.1)	5.1%(2.7–7.4)	8.3%(3.4–13.3)	0.200
Proportion with more than one sexual partner in the past 12 months (95% CI)	17.4(13.9–20.9)	18.3% (14.2–22.5)	15%(8.6–21.3)	0.411
Proportion never been pregnant before (95% CI)	53.8% (49.3–58.5)	58.4%(53.1–63.7)	40.8%(32.1–49.6)	0.001
Proportion with ≥1 miscarriage in the past (95% CI)	12.4%(9.2–15.5)	14.2%(10.4–18.1)	7.1%(2.4–11.9)	0.050
Proportion with ≥1 still birth in the past (95% CI)	1.1%(0.1–2.2)	1.3%(0.03–2.5)	0.9%(−0.8–2.6)	0.757
Proportion with ≥1 children dead (95% CI)	10.2%(7.3–13.1)	11.0%(7.6–14.4)	8.0%(3.0–13.1)	0.472
Proportion treated for STIs (95% CI)	22.9% (19.0–26.8)	25.5% (20.8–30.2)	15.0%(8.6–21.4)	0.018

There was a statistically significant difference between the median CD4 counts of the RI (440 cells/µL, IQR; 316–555 cells/µL) and those with LTI (374 cells/µL, IQR; 350–483 cells/µL) groups (*p* = 0.05).

Plasma HIV RNA viral load quantitations were performed on samples from the first 105 women enrolled into the study. Twenty-five of the 105 women were classified as RI and 80 had LTI. The mean viral load for the women with LTI (3.57 log_10_ RNA copies/ml, 95% CI: 3.57–3.94 log_10_ RNA copies/ml) was not significantly different (p = 0.251) from that of those with RI (3.58 log10 RNA copies/ml, 95% CI: 3.21–3.94 log10 RNA copies/ml).

The estimated time of conception was calculated for 96 of the 125 RI women (77%). Considering the estimated dates of conception and the probable timing of infection calculated using the cBED assay window period of 155 days, 93.9% (93) of the 96 women with evidence of RI and estimated conception dates were infected during the current pregnancy.

Genotypic drug resistance testing by population sequencing was conducted on the first 303 samples collected. Of these, 236 (78%) were successfully sequenced. Sixty seven (22%) were not amplifiable. The first 240 codons of the reverse transcriptase (RT) gene were sequenced from the 236 women (Genbank accession numbers: GQ463284-GQ463339 and HQ874283-HQ874432). One hundred and seventy five (74%) of these samples also yielded sequences from Protease (PR). On phylogetic analysis, all the sequences clustered with HIV-1 subtype C reference sequences; and were also classified as HIV-1 subtype C by the REGA subtyping tool on the Stanford HIVDB and confirmed by calibrated resistance programme (CPR). All the sequences were subtype C HIV-1. The PR and RT majority consensus sequence generated from the 175 and 236 sequences, respectively were 100% identical to the HIV-1 subtype C consensus sequences from the Los Alamos HIV database. Using HIVDB's calibrated resistance program and the Stanford Drug Resistance Mutation list (2008), two out of 236 (0.85%) sequenced samples had drug resistance mutations. The mutations present were PR I85V in Subtype C Drug Resistance (SCR) patient identity number 423 (SCR423), and RT Y181C in SCR541 selected by PIs and NNRTIs respectively. SCR423 was misclassified as RI, had exposure to SD NVP in 2004 for pMTCT of HIV whilst SCR541 had LTI.

**Figure 1:**
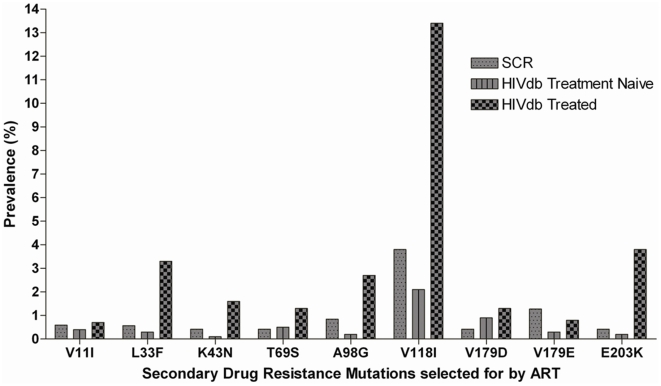
Secondary drug resistance mutation found in the study population Subtype C Drug Resistance (SCR) compared with data from treated and treatment naïve patients in the HIVDB.

The specimen with the Y181C mutation also had the nucleoside reverse transcriptase inhibitor (NRTI) selected mutation, T69A. Nineteen other samples (8%) had mutations (Figure 1) that may be selected by ARV drugs but have also been described in samples from treatment naïve patients with varying frequencies. In accordance with the WHO list of transmitted drug resistance mutations for population surveillance purposes, these secondary drug resistance mutations do not cause or contribute to drug resistance and therefore are not included as PDR mutations [26]. However, these mutations can potentially reduce drug susceptibility in association with other mutations. The frequencies of these mutations in our subtype C sequences were not statistically different from those of treatment naïve patients from the Stanford HIVDB. Some sequences contained recently described mutations that may reduce the susceptibility to etravirine; E28K (0.84%), V90I (0.42%) and E138Q (0.42%).

The PR and RT majority consensus sequence (50% majority rule) generated from the 175 and 236 sequences respectively were identical to the HIV-1 subtype C consensus sequences as defined by the Los Alamos HIVDB. The Maximum Likelihood (ML) tree generated from 227 sequences with the first 240 RT codons did not demonstrate specific clustering of the study sequences.

## Discussion

Genotypic drug resistance testing of samples from the 236 women demonstrated little evidence of transmitted or acquired drug resistance among these young, HIV positive pregnant women in Chitungwiza in 2006–2007. Surveillance for transmitted drug resistance is ideally conducted among recently infected individuals from longitudinal studies with estimated dates of infection. Here we sought to establish the prevalence of recent infection and identify PDR among young treatment naïve women attending antenatal clinics by including the BED assay to identify women who may have been recently infected. Young pregnant primigravida women are assumed to have acquired infection within the last few years and data on ART exposure was also collected as recommended by the WHO resistance network [20], [21], [26], [38].

The accuracy of cBED assays to estimate recent infection and incidence in diverse subtypes, populations and settings is controversial. In settings where HIV-1 subtype B predominates, the assay misclassifies only 2–3% of cases [29]. In Uganda, where subtypes A and D predominate, the cBED assay estimated an incidence rate of 6.1% and 6.0% in Masaka and Kakira districts respectively. However, prospective incidence rates in these same areas the previous year were 1.7% (Masaka) and 1.4% (Kakira) suggesting an overestimation of recent infection and misclassification by the assay [39]. In high HIV prevalence rural South Africa, where subtype C predominates, the cBED assay was shown to more accurately classify patients if locally measured long-term false positive ratio (FPR) are used but may underestimate incidence when used with FPR from other settings. FPR is a correction method to account for non-recently infected who are misclassified as recently infected. It is based on the assumption that after infection, there is finite time progression to cBED threshold except in non progressors. The fraction of HIV infected individuals who have been infected beyond the cBED threshold is the long- term FPR [30].

A prospective study conducted in Zimbabwe using specimens from pregnant women with known dates of seroconversion, recommended a cut off of 187 days [31] instead of the 155 days recommended by the cBED kit manufacturer. Studies from South Africa, Uganda and Zambia have used the 155 day cut off value [29], [30], [39], [40], [41], [42], [43]. In the current study, there was a modest decrease in the estimate of recent infection, using the 187 day ZVITAMBO study cut off, (data not shown). Thus to distinguish probable RI from LTI on a population basis, it would appear that either of the two cut-offs may be used.

There was no evidence of PDR in young pregnant ART naïve women with recent infection. The low prevalence of PDR in the studied population is consistent with other studies that have been conducted in settings that are currently scaling up ART in southern Africa [20], [21], [22]. These studies have reported prevalence levels of <5%, the WHO detection threshold limit in antiretroviral naïve populations. However, a higher frequency of PDR has recently been reported from a large study in Rwanda, South Africa, Uganda and Zambia where slightly more than 5% drug resistance was identified in samples collected from 2006 through 2009 [24]. Following scaling-up of ART, in resource poor countries, the WHO developed a protocol called HIV drug resistance threshold survey for use in the evaluation of the extent of transmitted HIVDR in ART-naive RI women which was to be used as a supplement to HIV sentinel serosurveys. In the WHO protocol, transmitted HIVDR is categorized into three groups; <5% (low HIVDR), 5–15% (Medium HIVDR) and >15% (High HIVDR). The <5% level is the desired threshold for any country scaling-up ART. Threshold assessment surveys are conducted to assess if drug resistant HIV is sufficiently prevalent in the country to indicate a need for sentinel surveillance. In our study, there was no evidence of PDR in the RI group. SCR423, the woman misclassified as RI by the cBED assay had exposure to SD NVP in a previous pregnancy, although the mutation she harbours is not associated with SD NVP.

There were several limitations encountered in this study. The prevalence of drug resistance mutations in the study population was very low, and thus there was no ability to estimate the frequency of transmitted drug resistance. However, the focus on young, largely primigravida pregnant women was expected to identify recent infections. The younger age and significantly higher CD4 cell numbers among those estimated to have recent infection provide some evidence for the veracity of the BED assays. Only a few of the women reported that they or their partners had been exposed to ART. Of the few women who were exposed to ART for pMTCT of HIV, one of them misclassified by the cBED assay as RI, had a drug resistant mutation. We were not able to obtain direct access to partners of these young women, men who were on average 8 years older than their wives or partners who may have been the source of transmitted infection. As estimated by the BED assay and estimated dates of conception, young pregnant women are at very high risk of acquiring infection around conception and early pregnancy. The high percentage of women infected during pregnancy shows the urgent need for more prevention education in young women, their spouses and partners.

There was a low sequencing rate for the sample in this study (78%), which may have been due to storage and shipping conditions. The demographics of the 22% of the women whose specimens could not be amplified for genotyping did not significantly differ from those with amplifiable specimens. Unfortunately not all samples had their viral load measured; this could have helped in ascertaining the cause of the low amplification rate.

The prevalence of PDR in Chitungwiza, four years after the commencement of the national ART program, is still far below the WHO threshold limit of 5%. To preserve the low prevalence of PDR, proper prescribing practices of ARV drugs, adherence to ART education should be maintained. Patients should be monitored to identify those failing therapy and provided alternative therapies, condom promotion and adherence counseling to prevent the spread of drug resistant viruses. As the ART coverage in Zimbabwe increases during the coming years, monitoring patients failing ART will be important to track the evolution of drug resistance in addition to continued surveillance for primary, transmitted drug resistance among drug naïve individuals.
